# Perceived and desired weight, weight related eating and exercising behaviours, and advice received from parents among thin, overweight, obese or normal weight Australian children and adolescents

**DOI:** 10.1186/1479-5868-8-68

**Published:** 2011-06-26

**Authors:** Jennifer A O'Dea, Nancy K Amy

**Affiliations:** 1 Faculty of Education & Social Work, Building A35, The University of Sydney NSW, 2006, Australia; 2 Department of Nutritional Sciences & Toxicology, Morgan Hall, MC 3104, University of California, Berkeley, USA

## Abstract

**Background:**

Thin children are less muscular, weaker, less active, and have lower performance in measures of physical fitness than their normal weight peers. Thin children are also more frequently subjected to teasing and stigmatization. Little is known about thin children's weight perceptions, desired weight and attitudes and behaviours towards food and exercise. The study aimed to compare perceived weight status, desired weight, eating and exercise behaviours and advice received from parents among thin, overweight, obese or normal weight Australian children and adolescents.

**Methods:**

The sample included 8550 school children aged 6 to 18 years selected from every state and territory of Australia. The children were weighed, measured and classified as thin, normal, overweight or obese using international standards. The main outcome measures were perceived and desired weight, weight related eating and exercising behaviours, and advice received from parents.

**Results:**

The distribution of weight status was - thin 4.4%; normal weight 70.7%; overweight 18.3%; and obese 6.6%. Thin children were significantly shorter than normal weight, overweight or obese children and they were also more likely to report regularly consuming meals and snacks. 57.4% of thin children, 83.1% of normal weight children, 63.7% of overweight and 38.3% of obese children perceived their weight as "about right". Of the thin children, 53.9% wanted to be heavier, 36.2% wanted to stay the same weight, and 9.8% wanted to weigh less. Thin children were significantly less likely than obese children to respond positively to statements such as "I am trying to get fitter" or "I need to get more exercise." Parents were significantly less likely to recommend exercise for thin children compared with other weight groups.

**Conclusions:**

Thin children, as well as those who are overweight or obese, are less likely than normal weight children to consider their weight "about right'. Thin children differ from children of other weights in that thin children are less likely to desire to get fitter or be encouraged to exercise. Both extremes of the spectrum of weight, from underweight to obese, may have serious health consequences for the individuals, as well as for public health policy. Health and wellness programs that promote positive social experiences and encourage exercise should include children of all sizes.

## Background

If good health in children were associated with the most desirable body type portrayed in popular media, one might expect thin children to be demonstrably healthier than their obese, overweight and perhaps even normal weight peers. Problems related to malnutrition and eating disorders in thin children have been studied, but persistent thinness that is not due to these causes has received little scrutiny.

Compared to normal weight children, thin children may have a range of significant health problems not directly related to malnutrition and eating disorders including: lower body fat stores; delayed growth; increased risk of osteoporosis; lower muscle and skeletal mass associated with low bone mineral content; and increased risk of bone fracture [1-7].

In studies of physical fitness, thin adolescents spent less time doing physical activity [8] and have lower performance in measures compared to normal weight adolescents [1,9]. There may be an *in utero *explanation for thinness in children that is associated with maternal under nutrition in that women with low pre-pregnancy weight are at increased risk of pre-term delivery, small-for-gestational age infants, and fetal and neonatal deaths [7,10,11]. Further, strong evidence exists for the genetic basis of thinness in humans and animals. Since underweight (as well as obese) adults suffer increased mortality relative to normal weight or overweight adults [12], thin children who become thin adults may be at risk of having shortened life spans. Recent reports indicate that underweight in adulthood is clearly associated with an increased mortality due to diabetes, kidney disease, respiratory disease, infectious disease, and other causes in the USA [13] and that these discrepancies are independent of smoking status.

Weight issues in children are important not only because of health risks, but also because of potential emotional consequences of practices such as stigmatization. Thin children are subjected to frequent teasing at a much higher rate than normal or overweight children and are also at a greater risk of unhealthy weight control behaviours [14]. Thin boys are more likely to dislike school and consider themselves to be poor students [15].

Studies that explore body image in children generally compare the attitudes of overweight and obese children with non-obese children, and the attitudes of thin children are not reported separately from other children. Little is known about thin children's body image, attitudes, and behaviours towards food and exercise.

Thinness is defined as low Body Mass Index (BMI) for age and gender in children. However, the prevalence of thinness across countries and years has been difficult to compare because underweight has been defined differently in these studies. To address this problem, Cole developed a common set of references for international comparisons [16]. Thinness in children and adolescents is defined based on age and gender specific curves that are linked to the World Health Organization (WHO) recommended adult cut-off points of BMI 16, 17, and 18.5 at age 18. These thinness standards allow distinctions between different grades of under nutrition and different levels of risk in children. The methods currently used worldwide to develop the thinness cut-offs are similar to those used by the International Obesity Task Force (IOTF) for overweight and obesity cut-offs [17] and therefore allow for the comparison of prevalence of thinness, overweight and obesity.

The objective of this study was to examine the relationships between perceived and desired weight, weight related eating and exercising behaviours, and advice received from parents among thin, overweight, obese or normal weight Australian children and adolescents.

The study measured: 1) the prevalence of thinness, normal weight, overweight and obesity in a large, representative population sample of Australian school children; 2) the differences in perceived and desired weight, dietary habits, exercise behaviours and concerns about weight between thin children and their peers of other sizes; and 3) the relationship between the parental advice given to children of different weight status and the children's' behaviours and attitudes concerning body size and exercise.

## Methods

Data in this study were collected as part of the Youth Cultures of Eating Study, a 3-year Australian Research Council funded study of health, weight, culture and eating among 8550 school children from every state and territory of Australia in 2006. The descriptions of study recruitment and sampling are given in previously published articles [18-20]. Children in grades 2 to 12 (aged 6 to18 years) participated, from randomly selected class groups within schools that had been randomly selected from lists of all state and territory's schools in Australia. The sample included students from public, private, and Catholic schools in both rural and urban areas, with low, middle and high socio-economic status (SES) levels. The ethnic distribution of the sample was representative of the general population and has been described previously [19]. The participation rate was high at 82%.

Children were weighed and measured using digital scales and portable stadiometers in light school uniforms, after removing jackets, shoes and emptying their pockets, in private areas of the schools, under the supervision of the first author and trained research assistants. The prevalence of normal weight, overweight and obesity was measured using the International Obesity Task Force (IOTF) BMI cut-offs, with age in years taken to the 0.5 years cut off [17]. Height was measured to the nearest 0.5 cm using a portable stadiometer. Weight was measured to the nearest 0.1 kg using portable digital scales. Participants' height and weight were used to calculate the age and sex-adjusted BMI percentile. Thinness, in grades 1, 2, and 3 children and adolescents was based on adult BMI of 16, 17 and 18.5 at 18 years, and curves provided age and sex-specific cut-off points [16]. Students were grouped by age into two categories: primary school students, aged 6 to 12 years; and secondary school students, aged 13 to18 years.

Details of the questionnaire and the study variables have been described previously [18-20]. The questionnaire collected demographic details of: gender; age; ethnic background; school SES (Low SES, Middle SES, High SES determined by government based on combined school indices of family income and parental education [18-20]; weight perceptions; desired weight; as well as other nutrition and exercise-related questions. The students completed the questionnaires under the supervision of the first author and research assistants during regular class times.

The assessments of the students' satisfaction with weight were made by evaluating answers to questions relating to whether the student thought that he/she was "about right", "too thin" or "too fat." Each student was also asked about his/her desired weight using a Likert scale of 1-5, as follows: "Would you like your weight to be a little lighter", "a lot lighter", "the same as present", "little heavier", or "a lot heavier". The nutritional quality of breakfast score was calculated [20] by asking students to report everything that they had eaten and drunk for breakfast on the morning of the study, and scoring the students' breakfasts on a scale from 0 to 10 with a high score indicating greater nutritional quality. A score of zero was given if nothing was consumed and a score of 10 was given for a breakfast consisting of a grain or cereal food group plus a meat/meat substitute protein food group plus a fruit/vegetable food group plus a dairy/calcium food plus a low fat selection; a common example of a breakfast with a score of 10 was fruit plus cereal and low fat milk.

The study was approved by the University of Sydney Human Ethics committee as well as each of the Departments of Education in every state and territory of Australia. Informed parental consent was required for each student to participate, and each student's verbal consent was obtained on the day of the study.

Descriptive chi-square analyses were undertaken to compare differences in body image and desired weight by gender and weight categories. Separate chi-square analyses were used to examine associations between the categorical variables and BMI groups. For post hoc analyses, these tests were followed by pairwise chi-square tests with Bonferroni's correction to indicate significant differences between groups. To assess differences in mean scores within groups and between groups, one-way analyses of variance (ANCOVA) were used, controlling for age as a covariate; Tukey's multiple range test was used for the post hoc test. A *p *value of < 0.05 was selected to determine statistical significance. Data analysis weighting methods were applied to adjust for complex sampling strategies. Sampling weights were calculated based on census data from 2006 and weightings were applied in all analyses and are described in a previous article (18).

## Results

### Participants

A description of the participants' mean age, Body Mass Index (BMI), and height by weight status grouping is given in Table 1. The distribution of weight status was as follows: thin 4.4%; normal weight 70.7%; overweight 18.3%; and obese 6.6%. Results about the prevalence of overweight and obesity are previously published [18,19]. When the sample was separated by gender, 3.8% of the boys (159/4157) and 5.0% of the girls (219/4393) were thin. The prevalence of thinness of grades 1, 2, and 3 (corresponding to BMI 16, 17 and 18.5 at 18 years respectively) in the overall sample was: grade 1 (0.14%, *n *= 12/8550, 3 boys, 9 girls, 0.01% in boys, 0.2% in girls); grade 2 (0.44%, *n *= 38/8550: 12 boys, 26 girls, 0.29% boys, 0.59% girls), and grade 3 (3.84%, *n *= 328/8550: 144 boys, 184 girls, 3.46% boys, 4.19% girls).

**Table 1:** Description of students' mean age, Body Mass Index (BMI), and height by weight status grouping.

	All	Thin	Normal	Overweight	Obese	F value	p value
% of Sample (%, n)	100 (8550)	4.4 (378)	70.7 (6040)	18.3 (1566)	6.6 (566)		
BMI in kg/m^2 ^(mean, SD)	20.4 (4.1)	15.1 (1.3)	19.0^a ^(2.2)	24.1^ab ^(2.3)	30.0^abc ^(4.1)	5661.75	***
Age in years (mean, SD)	12.8 (2.4)	12.8 (2.3)	12.8 (2.4)	12.9 (2.3)	12.8 (2.5)	1.17	NS
Height in cm (mean, SD)	155.4 (14.3)	151.6 (14.3)	154.6^a ^(14.5)	157.6^ab ^(13.3)	158.5^ab ^(13)	36.27	***
Boys height 6-12 yr (mean, SD)	146.4 (9.9)	141.9 (9.6)	144 ^a ^(10.6)	148.5^ab ^(10)	151^ab ^(9.4)	37.2	***
Boys height 13-18yr) (mean, SD)	168.4 (9.4)	163.6 (10.1)	168.9^a ^(9.4)	170.6^a ^(8.9)	170.6^a ^(9.1)	13.8	***
Girls height 6-12 yr (mean, SD)	145.0 (10.5)	140.3 (10.5)	143.8^a ^(10.7)	147.7^ab ^(10.7)	148.2^ab ^(10)	25.8	***
Girls height 13-18 yr (mean, SD)	162.1 (7.4)	160.5 (8.7)	161.9 ^a ^(6.9)	162.4^ab ^(6.6)	163.6^ab ^(7.5)	4.4	**

NS: Non significant **p < 0.01; ***p < 0.001

Statistical comparisons by ANCOVA controlling for age and with Tukey post hoc test.

^a^Significantly different from thin

^b^Significantly different from normal

^c^Significantly different from overweight.

Thin students were significantly shorter than students of other weight status (Table 1). Normal weight students were shorter than the overweight and obese students; but overweight and obese students did not differ significantly in height. The height of boys and girls did not differ in the 6 to 12 year old group, but boys in the 13 to 18 year old group were taller than the girls. Thin students were shorter than overweight and obese students in all the age and gender categories.

There were no significant differences in the proportion of thin children in the 6 to 12 year old group (4.39%, 186/4239) versus the 13 to 18 year old group (4.45%, 192/4311), or in the distribution of thin children throughout the three SES levels (p = 0.57). In contrast, the obese students were predominately from the low SES schools communities [21].

### Perceived and desired weight

Students were asked about their perceived weight and their desired weight. Results in Table 2 show that 57.4% of thin students considered themselves "about right" (51.6%, 82/159 of thin boys, 69.9%, 153/219 of thin girls). When compared with other weight status groups, the percentage of thin children considering themselves "about right" was lower than normal or overweight students, but not obese students (Table 2). Most (48.0%) thin students desired a weight that was "a little heavier" than their current weight. A matter of potential concern was that 9.3% of thin students (35/378, 10 boys, 25 girls) wanted to be "a little or a lot thinner". These students were evenly divided between the primary and secondary school age groups. In comparison, among normal weight students, 83% considered themselves "about right", and 29.7% of boys (708/2385) and 70.0% of girls (1677/2385) wanted to be "a little or a lot thinner".

**Table 2:** Comparison of perceived and desired weight among school students of different weight status.

	Thin	Normal	Overweight	Obese	**χ**^**2**^	p value
	% (n)	% (n)	% (n)	% (n)		
**Perceived weight**					1999.42	***
Too thin	42.0 (150)	8.5 (473)	0.8 (12)	1.2 (6)		
About right	57.4 (205)	83.1 (4632)	63.8 (925)	38.3 (194)		
Too fat	0.60 (2)	8.4 (470)	35.4 (514)	60.5 (306)		
**Desired Weight**					1686.39	***
A lot heavier	5.9 (21)	1.6 (90)	0.5 (7)	2.4 (12)		
A little heavier	48.0(171)	17.0 (947)	4.2 (67)	4.4 (22)		
Same as present	36.3 (129)	38.7 (2155)	15.0 (219)	9.1 (46)		
A little lighter	8.4(30)	36.7 (2049)	57.5(840)	44.7 (225)		
A lot lighter	1.4 (5)	6.0 (336)	22.8 (334)	39.4 (198)		

***p < 0.001

### Eating behaviour

The results given in Table 3 show that thin and normal weight students reported eating breakfast, lunch, and snacks significantly more frequently than overweight or obese students.

**Table 3:** Comparison of eating behaviours among school students of different weight status

	Thin	Normal	Overweight	Obese	**χ**^**2**^	p value
	% (n)	% (n)	% (n)	% (n)		
Usually eat breakfast	84.9 (304)	84.8 (4755)	82.1 (1200)	76.6^abc ^(386)	27.26	***
Usually morning snack	78.5 (281)	77.2 (4323)	72.7^b ^(1061)	68.3^ab ^(344)	31.08	***
Usually eat lunch	93.6 (335)	92.6 (5183)	90.8 (1326)	88.6^b ^(449)	14.76	**
Usually afternoon snack	88.8 (318)	89.0 (4982)	83.5^b ^(1218)	80.5^ab ^(405)	55.71	***
Usually eat dinner	96.7 (347)	98.0 (5495)	98.2 (1436)	98.2 (499)	3.6	NS
Usually pm snack	70.2 (250)	67.1 (3744)	53.4^ab ^(773)	50.9^ab ^(255)	136.8	***
Take vitamins	29.4 (105)	23.5 (1313)	20.7^a ^(301)	18.1^ab ^(92)	20.42	***

**p < 0.01 ***p < 0.001; NS Non significant

^a^Significantly different from thin

^b^Significantly different from normal

^c^Significantly different from overweight.

The percentage of students who ate breakfast decreased from 90.7% of primary school students (3302/3641) to 78.0% (3343/4288) of secondary school students. This decrease was seen prominently among obese secondary school students with only 68.5% (187/273) reporting that they ate breakfast on the day of the study.

The nutritional quality of breakfast score was significantly higher in thin students than obese students. [Breakfast scores (Mean ± SD): thin students 6.2 ± 2.7; normal weight students 6.2 ± 2.9; overweight students, 5.8 ± 2.9; obese students 5.2 ± 3.1; F = 19.98, df = 3, p < 0.001].

### Advice received from parents/carers about weight, food and exercise

Students of different weight status received different types of advice from parents and carers concerning their weight, nutrition and exercise. In many cases, boys and girls received markedly different advice.

A comparison of the advice concerning weight and food consumption given to students of different weight status, with the student's own beliefs is given in Table 4. Parents/carers advised 41.3% of thin students that they did "not eat enough"; 10.2% of thin students were advised that they "eat too much." About a third of the thin students (31.8%) reported that they were trying to "gain weight." In comparison, 24.6% of normal weight students reported that they were "dieting to lose weight." Most of the students from all the weight categories thought that they had "good eating habits."

**Table 4:** Comparison of advice received by students from parents/carers about their weight status and eating habits and students' corresponding beliefs and weight related behaviours.

	Thin	Normal	Overweight	Obese	**χ**^**2**^	p value
	% (n)	% (n)	% (n)	% (n)		
***Advice received from parents/carers - "I am told that I ....***						
Should lose weight	3.4 (12)	8.0 ^a ^(445)	30.2^ab ^(439)	51.8^abc ^(264)	1077.03	***
Do not eat enough	41.3 (148)	21.1^a ^(1183)	10.8^ab ^(158)	7.7^ab ^(39)	235.09	***
Eat too much	10.2 (36)	15.5^a ^(860)	26.6^ab ^(383)	42.8^abc ^(216)	299.83	***
***Beliefs & Behaviours***						
I have good eating habits	70.7 (251)	76.3 (4250)	68.0^b ^(979)	62.3^b ^(314)	78.81	***
I am currently trying to lose weight	4.5 (16)	22.6^a ^(1259)	56.9^ab ^(828)	74.8^abc ^(376)	1202.54	***
I am currently trying to gain weight	31.8 (114)	10.0^a ^(559)	2.6^ab ^(38)	2.6^ab ^(13)	324.71	***
I diet to lose weight	7.5 (26)	24.6^a ^(1360)	47.5^ab ^(689)	60.3^abc ^(302)	587.8	***
I diet to gain weight	21.7 (77)	7.5^a ^(403)	3.1^ab^(42)	5.4^a ^(25)	147.1	***
I eat more when I am bored or sad						
Boys	30.8 (46)	31.9 (872)	29.3 (202)	33.4 (93)	2.26	NS
Girls	55.8 (116)	52.7 (1505)	53.1 (406)	59.0 (135)	3.8	NS

*** p < 0.001

^a^Significantly different from thin

^b^Significantly different from normal

^c^Significantly different from overweight.

The advice given thin students concerning exercise and "building up their bodies" differed from that given to other students (Table 5). Over half of boys of all weights were advised to "build up their bodies," girls were advised less on this aspect, with thin girls higher than other groups (36.6%). A significantly lower percentage of thin students received advice to "do more exercise," than obese students (20.2% versus 62.9%). In this regard, the students' own attitudes reflected parental concern. The thin students were significantly less concerned about "increasing exercise" (42.7% versus. 84.7%) and "trying to get fitter" (57.5% versus 87.7%) than obese students. 50% of boys and 30% of girls of all weights reported currently trying to build up their bodies and muscles.

**Table 5:** Comparison of advice about exercise given to students by their parents and carers and the students corresponding attitudes and behaviours

	Thin	Normal	Overweight	Obese	**χ**^**2**^	p value
	% (n)	% (n)	% (n)	% (n)		
***Advice from parents and carers - "I am told that I ......"***						
Should build up my body						
Boys	32.9 (49)	25.4^a ^(675)	23.5^a ^(161)	31.5^bc ^(88)	11.98	**
Girls	26.8 (56)	12.5^a ^(357)	8.1^ab ^(63)	12.5^a ^(22)	55.01	***
Should do more exercise	20.2 (72)	28.5^a ^(1596)	48.8^ab ^(713)	62.9^abc ^(321)	437.68	***
Do too much exercise	9.5 (34)	12.5 (697)	8.0^b ^(116)	10.0 (51)	25.79	***
***Beliefs - "I feel that I ....."***						
Should develop my muscles						
Boys	74.5 (111)	77.0 (2098)	75.8 (519)	77.9 (218)	1.12	NS
Girls	46.1 (95)	44.4 (1267)	43.5 (336)	46.7 (106)	0.97	NS
Need to build up my body						
Boys	66.9 (99)	62.3 (1700)	60.9 (413)	66.4 (184)	3.81	NS
Girls	40.2 (82)	33.3 (947)	31.3 (240)	31.6 (71)	5.99	NS
Need to do more exercise	42.7 (152)	55.1^a ^(3068)	75.4^ab ^(1095)	84.7^abc ^(431)	379.12	***
Currently trying to build up my body						
Boys	54.4 (81)	59.5 (1619)	59.5 (405)	59.5 (165)	1.58	NS
Girls	36.6 (76)	27.7^a ^(788)	24.6^a ^(188)	22.9^a ^(52)	14.55	**
Currently trying to get fitter	57.5 (206)	70.8^a ^(3939)	83.2^ab ^(1205)	87.7^ab ^(443)	190.32	***

NS = not significant **p < 0.01 *** p < 0.001

^a^Significantly different from thin

^b^Significantly different from normal

^c^Significantly different from overweight.

## Discussion

The major aim of the current study was to investigate thinness in a representative sample of Australian school children and our findings suggest that approximately 4% of the sample of 8550 Australian schoolchildren was thin. This finding is identical to a similar national study [8] conducted in 2007 confirming the results reported in this paper. The thin students were distributed among the three SES levels. In contrast, the obese children were predominately from low SES school communities, as shown previously [18-21].

In European countries, the prevalence of thinness has been reported to range from 4.8% to 11.9% in girls, and 3.1% to 9% in boys [1,3,22-25] but the prevalence of thinness in these countries differs by gender, age of children and region. The prevalence of thinness has decreased in some countries where under-nutrition has been a problem [24,25], but has increased in regions where there is a general desire for thinness. In some regions the rate of thinness in girls is concerning because it exceeds the rate of obesity [1,3,25]. Our study differs from those reported above in that the children were sampled from a wide diversity of regions in Australia and included children aged from 6 to 18 years.

Thin children displayed smaller stature than normal weight children. Other studies have shown that height is related to body weight and that obese children may be taller than normal weight children [4,5,8,26-28]. Thin children tend to be characterized by not only a lower percentage of body fat, but also by lower muscle mass and lower lean body mass [3]. Thinness due to under nutrition may result from food fussiness, increased satiety responsiveness [29] or fear of fatness leading to disordered eating. Bulik and Allison [30] present arguments for the genetic basis of thinness in humans and animals.

The results of the current study highlight several areas of concern regarding thin children. First, 12% of thin girls and 7% of thin boys said that they wanted to be a little or a lot lighter and these students were also dieting to lose weight. Some thin children were told that they "should lose weight" or that they "eat too much." The role of parental comments is important for influencing disordered eating and body dissatisfaction [31-33] and this may be an explanation for our current findings. Interestingly, whilst the questionnaire used in the study was not designed to diagnose clinical eating disorders, the findings may serve this purpose.

In one study of 14-year-olds, Allen et al. [32] estimated that 6% of the sample met full or partial criteria for a DSM-IV eating disorder and an additional 3% were at risk for developing an eating disorder. Being female and being perceived as overweight by the child's parents were the strongest predictors for eating problems in this study. This is of particular concern because dieting, body image dissatisfaction and teasing are all known predictors of eating disorders [34]. On the other hand, 41.3% of thin students in this survey reported that parents and peers told them that they "did not eat enough." Thin students in the current study were much less concerned than their peers about the need to "get fitter" or to "do more exercise," with only 20.2% being advised to do so. The findings of the current study warrant further investigation using a longitudinal study design in order to further clarify if some thin children deliberately engage in weight loss behaviours that may be triggered by parental advice.

The health benefits of physical activity for children are supported by many studies [35,36]. There is no evidence that thin children exercise more or are more fit than other children, and a recent study [8] showed that thin adolescents are less active than their normal weight peers. In contrast, participation in physical activity would be particularly important for thin children to overcome potential problems related to their low muscle mass and decreased muscle strength [1] and sport and physical activity are known to be important in the development of child and adolescent self-esteem [37] and positive adult body image [38]. Parental encouragement of physical activity is necessary for thin children, as children are more likely to have higher levels of physical activity when their parents and others encourage and support their participation [34,39,40].

Exercise in childhood is predictive of adult levels of exercise [40]. Low physical activity and poor cardiovascular fitness are independent predictors of mortality related to type 2 diabetes and chronic disease in general [41]. In fact, thinness is related to increased mortality and morbidity compared to normal and overweight (but not obese) adults [12,13]. The negative attitudes of thin students concerning exercise identified in the current study, if continued into adolescence and adulthood, do not bode well for a healthy population.

Finally, the findings of this study suggest that thin children may be at risk of body image problems, as only 57.4% of thin students considered themselves to be "about right" compared with 83.1% of normal weight students and 63.7% of overweight students. Children can develop body image concerns at very young ages [42]. Underweight as well as overweight youth reported higher levels of teasing than normal weight youth, and both groups face stereotypical attitudes and prejudices [43]. Weight teasing has been significantly associated with disordered eating behaviours in a large study from the USA [14] and thin children were found in another US study to dislike school and consider themselves to be poor students [15]. Thus, thin children (as well as children of all sizes) need to be considered and included in health promotion initiatives for self-esteem and positive body image.

This study has strengths in its large, generalized sample size, high participation rate, and accurately measured height and weight data, but has limitations in that there are no direct measures of caloric consumption or physical activity. Further research using qualitative interviews may better explain some of the findings of the current study. Of particular interest would be the attitudes of children and parents from various cultural and ethnic groups. Longitudinal studies could also further explore these issues over time, encompassing puberty and such studies could also incorporate actual health outcomes.

## Conclusions

Thin children, as well as those who are overweight or obese, are less likely than normal weight children to consider their weight 'about right'. Thin children differ from children of other weights in that thin children are less likely to desire to get fitter or be encouraged to exercise. Children of all body weights, from underweight to obese, are likely to experience health concerns. The issue of weight in children and adolescents has implications for public health policy. Health and wellness programs that promote positive social experiences and encourage physical activity should be implemented and include children of all sizes.

## Competing interests

The authors declare that they have no competing interests.

## Authors' contributions

JO'D designed the Youth Cultures of Eating Study, acquired the data, participated in study discussions, and helped to revise the manuscript. NKA designed the current analysis of the data, performed the statistical analyses, and wrote the manuscript. Both authors have read and approved the final manuscript.
